# Project Rosetta: a childhood social, emotional, and behavioral developmental feature mapping

**DOI:** 10.1186/s13326-021-00242-4

**Published:** 2021-04-15

**Authors:** Alyson Maslowski, Halim Abbas, Kelley Abrams, Sharief Taraman, Ford Garberson, Susan Segar

**Affiliations:** Cognoa, Inc., Palo Alto, CA USA

**Keywords:** Semantic hierarchy, Child behavior, Assessment, Autism spectrum disorder, Attention-deficit/hyperactivity disorder

## Abstract

**Background:**

A wide array of existing instruments are commonly used to assess childhood behavior and development for the evaluation of social, emotional and behavioral disorders such as Autism Spectrum Disorder (ASD), attention-deficit/hyperactivity disorder (ADHD), and anxiety. Many of these instruments either focus on one diagnostic category or encompass a broad set of childhood behaviors. We analyze a wide range of standardized behavioral instruments and identify a comprehensive, structured semantic hierarchical grouping of child behavioral observational features. We use the hierarchy to create Rosetta: a new set of behavioral assessment questions, designed to be minimal yet comprehensive in its coverage of clinically relevant behaviors. We maintain a full mapping from every functional feature in every covered instrument to a corresponding question in Rosetta.

**Results:**

In all, 209 Rosetta questions are shown to cover all the behavioral concepts targeted in the eight existing standardized instruments.

**Conclusion:**

The resulting hierarchy can be used to create more concise instruments across various ages and conditions, as well as create more robust overlapping datasets for both clinical and research use.

## Background

According to the 2011–12 National Survey of Children’s Health, researchers found that approximately 1 out of 7 U.S. children aged 2 to 8 years were reported to have a diagnosed mental, behavioral, or developmental disorder [1]. Identifying and addressing these concerns is of great importance so that interventions can start as early as possible when they have the greatest potential for improved lifelong outcomes. There are many instruments available to clinicians for the early assessment of mental, behavioral, or developmental disorders. However, many of these tools are widely used for predicting caseness, i.e., to identify individuals who are at high risk of having at least one psychiatric disorder, while others are primarily targeting a specific disorder. This presents many challenges for clinicians regarding which tools to use for making diagnostic decisions and identifying significant effects for appropriate therapeutic interventions [2].

The undertaking of project Rosetta was to address these challenges by creating a comprehensive semantic hierarchical grouping of concepts that have diagnostic relevance for child behavioral conditions, which can be used as a resource for child mental, behavioral, and developmental health diagnosis and treatment. We think of the importance of the Rosetta stone as more than just the historic object. “Rosetta” refers to the foundational revelation that communication could have multiple forms–oral with disparate languages, written with disparate characters, and the idea that each form of communication has a code that can be learned to promote understanding. It’s translation, but much more than translation. The outcome of this semantic hierarchical grouping of concepts can be used to create more concise instruments through the creation of Rosetta questions and answers that are representative of a single concept within the hierarchy that is translated from many potential input instruments and questions. This would allow for the identification of maximally predictive minimal subsets of features associated with each behavior by translating many questions to a single question regarding a single concept. Rosetta will then be able to be used as a framework for joining disparate data to create a virtual diagnostic instrument that covers more patients in a uniform way by having overlap between existing instruments and corresponding mappings that was not present before. This will make it possible to build improved machine learning algorithms that require dense datasets for creating innovative diagnostic tools.

The first generation of project Rosetta covered the functional elements of eight instruments, as shown in Table 1. These were chosen because they cover a wide range of child behaviors and diagnoses and were identified by subject matter experts to create a robust mapping. Some of these instruments are relatively time-consuming assessments that have to be completed by trained professionals, whereas others are shorter rating scales that have parent-, teacher-, and self-report forms. The range of ages for these instruments extends from 18 months up to adulthood, with some of them split into different versions based on age groupings. The time to complete the assessments can range from 5 minutes for a simple parental questionnaire up to 150 minutes for a more complicated assessment given by a trained professional, such as the Autism Diagnostic Interview-Revised (ADI-R). The number of questions in an assessment can range anywhere from 28 questions up to 192 questions, as in the Behavior Assessment for Children, Third Edition (BASC-3).

**Table 1:** Child Behavioral Instruments

Instrument	Age Range	Completion Time	Reporter(s)	Number of Questions	Conditions Covered
ADI-R [4]	2 years–Adult	90–150 min.	Clinician	93	Autism
ADOS-2 [5]	12 months–Adult	40–60 min.	Clinician	28–38	Autism
BASC-3 [6]	2–25 years	10–30 min.	Parent	105–192	Autism, ADHD, Anxiety,
			Teacher		Conduct Disorder, and
			Self		Depressive Disorders
BRIEF 2 [7]	5–18 years	5–30 min.	Parent	55–63	Autism, ADHD, Learning
			Teacher		disabilities, and other
			Self		acquired neurological
					conditions
CBCL [8]	1.5–18 years	10–30 min.	Parent	100–113	ADHD, Anxiety, Conduct
			Teacher		Problems, Depression,
			Self		Oppositional Defiant, and
					Somatic Problems
Conners 3 [9]	6–18 years	5–20 min.	Parent	99–115	ADHD, Conduct Disorder,
			Teacher		and Oppositional Defiant
			Self		Disorder
SRS-2 [10]	2.5 years	15–20 min.	Parent	65	Autism
	- Adult		Teacher		
			Self		
VADRS [11]	6–12 years	5–20 min.	Parent	43–55	ADHD, Anxiety, Conduct
			Teacher		Disorder, Depression, and
					Oppositional Defiant Disorder

The ADI-R is a standardized, semi-structured investigator-based interview for caregivers of children and adults for whom autism is a possible diagnosis, which provides a diagnostic algorithm for the International Classification of Disease (ICD), tenth edition definition of autism and the Diagnostic and Statistical Manual of Mental Disorders, fourth edition (DSM-IV). It includes 93 questions focusing on Early Development, Language and Communication, Reciprocal Social Interactions, and Restricted, Repetitive Behaviors and Interests [3].

The Autism Diagnostic Observation Schedule, Second Edition (ADOS-2) is a standardized protocol for the observation of social and communicative behaviors of persons with autism and related disorders. The instrument consists of a series of structured and semistructured prompts for interaction, accompanied by the coding of specific target behaviors associated with particular tasks and by general ratings of the quality of behaviors. The ADOS-2 consists of four modules so that each one is appropriate for children and adults at different developmental and language levels [4].

The BASC-3 is a norm-referenced diagnostic tool that uses a multi-dimensional approach to assess the behavior and self-perceptions of children and young adults ages 2 through 25 years. The BASC-3 includes teacher and parent rating scales separated into three forms: preschool, child and adolescent, as well as a self-report of personality separated into four forms: interview, child, adolescent, and college. It includes 23 clinical, adaptive and content scales, as well as ten clinical and executive functioning indexes and five composite scores that can be used to assist with differential diagnoses when used in conjunction with the Diagnostic and Statistical Manual of Mental Disorders, fifth edition (DSM-V) [5].

The Behavior Rating Inventory of Executive Function, Second Edition (BRIEF2) is an informant-report rating scale designed to assess executive behaviors in children and adolescents. It consists of nine domains of executive functioning, combined into three summary indexes, including the Behavioral Regulation Index (BRI), the Emotional Regulation Index (ERI), and the Cognitive Regulation Index (CRI). The BRI captures the child’s ability to regulate and monitor behavior effectively, while the ERI represents the child’s ability to regulate emotional responses and to shift set or adjust to changes in the environment, people, or plans. The CRI then reflects the child’s ability to control and manage cognitive processes and to problem solve effectively. The BRIEF2 can be used in conjunction with other rating scales, clinical interviews and observations to diagnose children and adolescents who have either developmental or acquired neurological conditions, such as learning disabilities, attention disorders, traumatic brain injuries, and other medical conditions [6].

The Child Behavior Checklist (CBCL) is a parent-report questionnaire for evaluating maladaptive behavioral and emotional problems in children and adolescents aged 2 to 18. It assesses a wide-range of internalizing behaviors, such as anxiety and depression, as well as externalizing behaviors, such as aggression and hyperactivity. When used in conjunction with the other rating scales within the Achenbach System of Empirically Based Assessment (ASEBA), the teacher-report and self-report questionnaires, it can be used to assess six DSM-V diagnostic categories, including Depressive Problems, Attention Deficit/Hyperactivity Problems, Anxiety Problems, Oppositional Defiant Problems, Somatic Problems, and Conduct Problems [7].

The Conners, 3rd Edition (Conners 3) is a thorough assessment of ADHD and its most commonly associated problems and disorders in school-aged youth. It is a multi-informant assessment with forms for parents, teachers, and youth. The assessment features multiple content scales that assess ADHD-related concerns as well as related problems in executive functioning, learning, aggression, and peer/family relations. In addition to these content scales, Conners 3 has five DSM-IV Symptom Scales that can be used as diagnostic criteria for ADHD and common comorbid disorders, including ADHD Inattentive, ADHD Hyperactive-Impulsive, ADHD Combined, Conduct Disorder, and Oppositional Defiant Disorder scales [8].

The Social Responsiveness Scale-Second Edition (SRS-2) is a 65-question rating scale measuring deficits in social behavior associated with ASD, as outlined by the DSM-IV. The SRS-2 consists of four rating forms across three age ranges, including parent-, teacher-, and self-report forms. There are five treatment sub-scales, including Social Awareness, Social Cognition, Social Communication, Social Motivation, and Restricted Interests and Repetitive Behavior, as well as an overall total score that are used to assess ASD [9].

The American Academy of Pediatrics (AAP) and the National Initiative for Children’s Healthcare Quality (NICHQ) jointly published the Vanderbilt ADHD Diagnostic Rating Scale (VADRS) as a psychological assessment toolkit to be used in the assessment and treatment of ADHD in a primary care setting. It includes versions specific for parents and teachers. In addition to questions corresponding to the ADHD diagnostic criteria of the DSM-IV, the VADRS includes symptom screens for four common comorbidities: oppositional defiant disorder, conduct disorder, anxiety, and depression [10].

## Methods

We set out to build the first generation of project Rosetta to include a minimal set of unique Rosetta questions representative of all of the behavioral, emotional, and developmental concepts identified within the mapping. Eight child behavioral instruments (listed in Table 1) were included in the analysis that led to the creation of the behavioral mapping underlying Rosetta. The process for creating Rosetta involved analyzing the existing child behavioral instruments, developing a semantic hierarchy of clinical domains that are diagnostically relevant for childhood behavioral disorders, creating new Rosetta questions and answer choices, and mapping existing instrument concepts to Rosetta questions and answers. The process for building Rosetta is detailed in this section.

### Procedure

#### Analysis of instruments

The first step of this process involved creating a document to analyze each of the eight child behavioral instruments listed in Table 1. All versions of each instrument by age group were analyzed, whereas only the parental version of an instrument was analyzed if there were multiple forms for different raters. A uniform set of column names was used for all instruments, including item and subject, to compare the concepts being asked in each instrument to create a comprehensive child behavioral mapping.

#### Clinical domain categorization

**Fig. 1:**
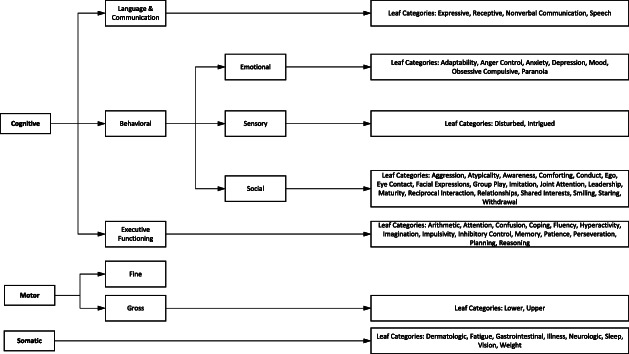
Semantic Hierarchy of Clinical Domains. The hierarchical scheme that was created for categorizing each of the clinical instrument subjects into a mapping of clinical domains. The hierarchy shown includes all of the top-level domains, sub-groupings, and leaf categories within the Rosetta mapping

The subjects from the instruments that were analyzed in the previous step were added to an aggregate tab within the document. This made it easier to examine each subject to determine which concepts within these instruments had overlapping semantic meaning. A scheme was created to use for categorizing each of the subjects into a mapping of clinical domains, shown in Fig. 1. A team of subject matter experts which included clinical neuropsychology, developmental psychology, and pediatric neurology representations were consulted in order to arrive at groupings and sub-groupings within the mapping. At the top level of the hierarchy, there were three broad domains, including Cognitive, Motor, and Somatic, which were then further broken down into a total of 57 leaf categories.

The cognitive grouping encompasses all functions of the mind, such as thought, perception, and the organization of information and ideas [11]. This top level group was further broken down into 3 sub-groups, Behavioral, Language & Communication, and Executive Functioning. The Behavioral sub-group was then broken down into 3 more sub-groups, Emotional, which was broken down into 7 leaf categories; Sensory, which was broken down into 2 leaf categories; and Social, which was broken down into 19 leaf categories. Language & Communication was then broken down into 4 leaf categories, and Executive Functioning was broken down into 14 leaf categories. The second top level group was motor, which refers to the underlying factors involving the movement of the body [12]. This was then broken down into Fine and Gross motor, with gross motor being broken down into 2 further leaf categories. The last of the top level groupings was somatic, and this refers to those subjects associated with bodily responses [13]. Somatic was then broken down into 8 leaf categories.

Following the creation of the mapping, each instrument subject was assigned to a leaf category within the mapping in order to better understand the specific concepts being asked in each domain.

#### Rosetta question creation

Following the categorization of subjects, each leaf category was analyzed to determine all of the concepts that needed to be assessed by the creation of a novel single Rosetta question. As the concepts were analyzed by leaf category, Rosetta questions were phrased to ensure a minimal loss of ability to assess each particular child behavior. Subject matter experts drafted de novo questions based on the features identified (i.e., the broad clinical domain and leaf categories). Question versions were then reviewed to arrive at a final wording that was novel and concise yet well representative of the key behavioral concept underlying the overlapping subjects from all covered behavioral instruments. As an example of this iterative process, we looked at the Adaptability leaf category and found there were 32 subjects within this leaf from six different instruments. A subset of these subjects are shown in Table 2, to illustrate how well they overlap between instruments.

**Table 2:** Examples of Subjects Within the Adaptability Leaf Category

Instrument	Question Number	Subject
ADI-R	74	Changes in routine/schedule
ADI-R	75	Changes around the house
BASC-3 (Preschool)	88	Changes in surroundings
BASC-3 (Child)	47	Changes to schedule
BASC-3 (Adolescent)	156	Changes at school
BRIEF 2	11	Changes to situations
CBCL	21	Changes in routine
SRS-2	24	Changes in routine

Based on the semantic concepts represented in Table 2, a single Rosetta question was created by a team of subject matter experts to assess a child’s ability to adapt to changes to a routine, schedule or the environment. Again, we utilized a process of experts phrasing a de novo question followed by a team review and final phrasing. This particular Rosetta question was phrased as follows: “Does [NAME] become unusually upset with or have difficulty accepting small changes? For example, a change in [his/her] bedtime routine, weekly scheduled activities, or furniture arrangement in the house.”

#### Rosetta question mapping

A mapping was then created in a separate tab in the document so that each of the original instrument subjects were mapped to a corresponding Rosetta question. There was a single column that was created to include this mapping of all existing instrument subjects to a single Rosetta question. This mapping could then be used to convert any incoming dataset from a single existing instrument included within the mapping to a mapped Rosetta-specific dataset. As shown in the example above, all of the sample subjects in Table 2 could be mapped to the Rosetta question about a child’s ability to adapt to changes. Further details of the overlap created by carrying out this process are discussed in the “Results” section.

#### Rosetta question answer creation and mapping

As multiple subjects mapped to a single Rosetta question, each of these subjects tended to have varying types of responses. The ADI-R and ADOS-2 generally have descriptive answer choices that relate to the quality of behavior being assessed, whereas the remaining assessments have answer choices on a Likert scale referring to the frequency of that behavior. For the last step in this process, these differences had to be consolidated to create a new, consistent coding of answer choices that each of the subject responses could be mapped to, retaining as much of the function of the response nuances as possible.

In the example discussed in the section above, five different instruments were mapped to the question about adaptability to change and each of them was asked in a slightly different way with different answer choices. The corresponding ADI-R subjects had four descriptive answer choices, whereas the BRIEF2 and CBCL had three answer choices on a frequency scale, and the BASC-3 and SRS-2 had four answer choices on a frequency scale, shown in Table 3.

**Table 3:** Examples of Answer Choices for Subjects Mapped to Routine Change Question

Instrument	Answer 1	Answer 2	Answer 3	Answer 4
ADI-R	No difficulties with changes	Negative reactions to changes	Negative reactions to changes that cause distress	Resistance to changes that affects daily life
BASC-3	Never	Sometimes	Often	Almost Always
BRIEF2	Never	Sometimes	Often	
CBCL	Not True	Somewhat True	Very True	
SRS-2	Not True	Sometimes True	Often True	Almost Always True

The subject matter experts crafted de novo answer choices for Rosetta questions such that, where appropriate, descriptive quality-based responses were combined with the frequency responses typical of instruments like BASC-3 and BRIEF2. The subject matter experts were given the number of responses required for a particular question, as well as the type of responses required based on the instrument subjects that were being mapped to each Rosetta question. When subjects from BRIEF2 or CBCL mapped to a Rosetta question, three answer codes were created in Rosetta because that was the least amount of responses that would potentially be mapped if the child only had the BRIEF2 or CBCL instruments assessed. This was decided because it could not be inferred how a parent would have responded if given more answer choices. The new answer choices for this particular question that combined frequency and quality were as follows: 1=Rarely or never; 2=Sometimes, but with little interference in family life; and 3=Often, and with some interference with family life.

**Fig. 2:**
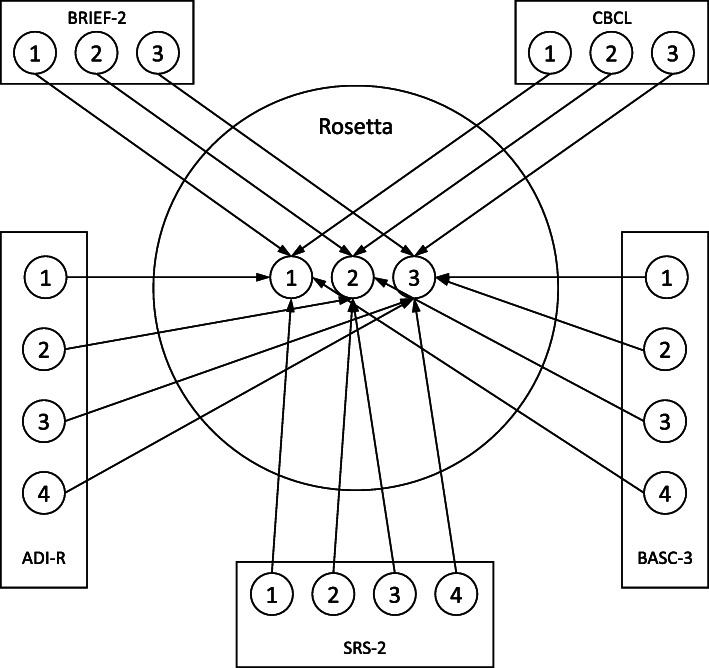
Example of Answer Choice Mapping for Adaptability, Routine Change question. Sample answer codes from existing instruments shown in Table 3 are in the boxes surrounding the Rosetta answer choices in the center circle. The mapping of each existing instrument answer choice to the corresponding Rosetta answer choice is shown by the arrow

We then mapped each of the existing instrument’s answer choices to the Rosetta answer codes based on how the subjects and answer choices overlapped with the phrasing of the Rosetta question and answer codes as shown in Fig. 2. Based on the phrasing of the Rosetta question and answers, the subjects from BRIEF-2 and CBCL had answer choices that were easily mapped one-to-one to the Rosetta answer choices. However, the other three instruments resulted in a slightly more complicated mapping of answer choices. The ADI-R had four descriptive answer choices to be mapped to the three Rosetta answer choices. Based on the severity of the answer choices in the ADI-R, the first two answer choices were mapped one-to-one with the Rosetta answer choices and the two most severe answer choices in the ADI-R were mapped to the most severe Rosetta answer choice with a code of 3. A similar process was used to map the four answer choices when mapping the SRS-2 and BASC-3 to the three Rosetta answer choices.

## Results

The final Rosetta document mapped existing behavioral instruments subject matters to 209 Rosetta questions. Table 4 shows the resulting fusion of subjects from the eight existing instruments that were included in the first generation of the Rosetta mapping.This table shows the number of existing instruments with overlap, the total existing instrument subjects that map to each leaf category, and the resulting number of novel Rosetta questions created within each leaf category in the mapping. On average, three instruments and seven instrument subjects mapped to a single Rosetta question.

**Table 4:** Resulting Fusion of Instrument Subjects within each Leaf Category

Base Category	Leaf Category	Overlapping Existing Instruments	Overlapping Instrument Subjects	Resulting Rosetta Questions
Cognitive,	Adaptability	6	32	4
Behavioral,	Anger Control	6	48	5
Emotional	Anxiety	7	126	17
	Depression	4	38	5
	Mood	4	16	3
	Obsessive Compulsive	4	15	4
	Paranoia	3	6	1
	Emotional	5	35	8
Cognitive,	Disturbed	2	4	1
Behavioral,	Intrigued	3	6	1
Sensory	Sensory	3	16	3
Cognitive,	Aggression	6	57	3
Behavioral,	Atypicality	3	32	6
Social	Awareness	8	52	11
	Comforting	3	5	1
	Conduct	4	100	14
	Ego	1	2	1
	Eye Contact	5	11	1
	Group Play	4	10	2
	Imitation	2	3	1
	Joint Attention	3	22	3
	Leadership	1	7	1
	Maturity	1	3	1
	Reciprocal Interactions	2	22	2
	Relationships	4	32	4
	Shared Interests	4	16	4
	Smile	2	2	1
	Staring	3	7	1
	Withdrawal	5	51	4
	Social	7	32	8
Cognitive,	Arithmetic	1	1	1
Executive	Attention	6	62	7
Functioning	Confusion	2	2	1
	Coping	1	9	1
	Fluency	2	6	3
	Hyperactivity	7	34	4
	Imagination	3	11	1
	Impulsivity	5	20	3
	Inhibitory Control	3	7	2
	Memory	5	14	3
	Patience	4	7	1
	Perseveration	5	18	1
	Planning	4	24	4
	Reasoning	4	19	5
	Executive Functioning	6	23	9
Cognitive,	Expressive	4	77	12
Language &	Nonverbal	2	10	2
Communication	Receptive	6	17	5
	Speech	4	12	3
Motor	Fine	2	8	1
	Gross	4	11	4

**Table 4:** Resulting Fusion of Instrument Subjects within each Leaf Category (*Continued*)

Base Category	Leaf Category	Overlapping Existing Instruments	Overlapping Instrument Subjects	Resulting Rosetta Questions
Somatic	Dermatologic	1	2	1
	Fatigue	3	8	1
	Gastrointestinal	2	18	2
	Illness	2	25	2
	Neurologic	2	10	2
	Sleep	1	7	2
	Vision	1	2	1
	Weight	1	1	1
	Somatic	1	2	1

As discussed in the example in “Methods” sections, a single novel Rosetta question was created within the adaptability leaf category to cover a child’s ability to adapt to changes, and twenty-one instrument subjects were mapped to this Rosetta question. Three additional Rosetta questions were created within this leaf category to assess other specific child behaviors associated with adaptability, and the remaining eleven instrument subjects were mapped accordingly. The thirty-two instrument subjects that were mapped to the four Rosetta questions came from six existing behavioral instruments, including ADI-R, BASC-3, BRIEF2, CBCL, Conners 3, and SRS-2. This mapping process led to a many-to-one mapping, where many subjects from different existing instruments mapped to a single Rosetta question.

**Fig. 3:**
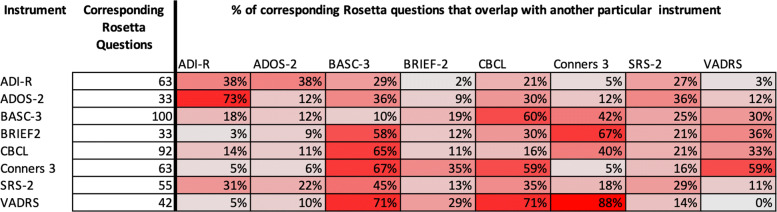
Heat map showing the overlap between instruments for all Rosetta questions. Instruments that have few overlapping Rosetta questions with the corresponding instrument in the column will be in the darkest orange, while instruments that have the most overlapping Rosetta questions with the corresponding instrument in the column will be in the darkest blue

The resulting overlap between existing instruments subjects and Rosetta questions is illustrated in the heat map in Fig. 3. For each existing instrument, the heat map shows how many Rosetta questions have overlap with every other instrument in the mapping. The diagonal from top left to bottom right shows how many Rosetta questions only map to one instrument. It can be seen from this that the ADI-R, CBCL, and SRS-2 have a considerable amount of subject matters that are difficult to find overlap with other existing questions. The last column in this figure shows the resulting number of Rosetta questions that overlap with each covered instrument.

### Case study: machine-learning-based, concurrent assessment of children for autism and ADHD

To demonstrate the utility of Rosetta as a platform to enable the development of simultaneous multi-condition assessment algorithms, we trained and validated a machine-learning algorithm to assess young children for the risk of autism or ADHD using a single questionnaire comprised entirely of Rosetta questions. The input data for this case study consisted of 3,731 patient records of children aged 4–10, each of whom underwent one or more of the clinical assessment instruments analyzed in the Rosetta project. The diagnostic labels for the dataset were assigned by licensed medical professionals, and the breakdown was 2,941 positive for autism, 343 positive for ADHD, and 447 negative for both.

As is the case in most clinical data collection settings, no single assessment instrument has been undergone by all patients in the dataset for this case study. Rather, the data covers 6 different Rosetta-friendly clinical assessment instruments with little overlap. Because many instruments have multiple versions, the total number of unique instrument versions was 15. Under traditional settings, it would not be possible to proceed with machine learning training in these conditions. However, with Rosetta available, it was possible to leverage the entire dataset as training and cross-validation samples to a machine-learning predictive algorithm.

The assessment algorithm identifies autism and ADHD using the Rosetta dataset as follows: first, a data imputation technique is used to infer values for missing Rosetta questions for every sample as needed, then machine learning was run to predict ADHD and autism. The imputation procedure was carried out in a custom manner as follows. For each feature containing missing values ensembles of random forests were trained to predict the missing values. For each iteration in the ensemble random subsets of candidate features were selected with which to do the imputation and only samples for which 100% coverage in the candidate features was observed were used in the training and in the inference. Random selections for which fewer than 50 samples of any labeled class were observed were discarded. For some samples it was not possible to impute missing values in certain features due to this minimum requirement. The procedure repeated until at least ten predictions for every sample were determined, and the results for each sample were averaged. This procedure was repeated for every feature with missing values. The advantage of this procedure is that it avoids cascading errors in multiple imputation-based procedures and the ensembling makes it robust against biases resulting from any particular inputs in certain feature columns.

After imputation was completed, a gradient boosted decision tree algorithm was trained to identify if either of autism or ADHD is present for a child. A second gradient boosted decision tree algorithm was trained to identify which of the two conditions is present, and was only used for predictions if the first algorithm identifies a child as having autism or ADHD. To train each algorithm an iterative procedure was used to identify the most predictive Rosetta questions to be used in model training. The training process made use of both the feature importances as determined by decision trees and the coverage of questions with valid values after imputation to prune the worst rows and the worst feature columns one at a time until expected cross-validated performances hit a maximum. The training process identified a total of 30 Rosetta questions as the relevant features for the assessment of autism and ADHD in a single questionnaire, as shown in Table 5.

**Table 5:** The 30 Most Relevant Rosetta Questions for the Assessment of Autism and ADHD Resulting from the Machine Learning Case Study

Rosetta Question ID	Rosetta Question Subject
Cognoa_Cognitive_Behavioral_Emotional_AngerControl_CalmingDown	Child is difficult to calm when upset
Cognoa_Cognitive_Behavioral_Emotional_AnxietyInternalization_Worry	Child tends to be worrisome
Cognoa_Cognitive_Behavioral_Sensory_Intrigued	Child has an unusual interest in certain sensory stimuli
Cognoa_Cognitive_Behavioral_Social_Atypicality_OddBehavior	Others seem to think the child acts strangely
Cognoa_Cognitive_Behavioral_Social_Atypicality_OddInteractions	Child has awkward interactions with others
Cognoa_Cognitive_Behavioral_Social_Atypicality_OutOfStepWithOthers	Child does not care about relating to others
Cognoa_Cognitive_Behavioral_Social_Awareness_GetsTakenAdvantageOf	Child has difficulty recognizing manipulative behavior
Cognoa_Cognitive_Behavioral_Social_Awareness_SenseOfHumor	Child understands humor
Cognoa_Cognitive_Behavioral_Social_Awareness_Unfair	Child has difficulty understanding fairness
Cognoa_Cognitive_Behavioral_Social_GroupPlay	Child participates in group play
Cognoa_Cognitive_Behavioral_Social_SharedInterests_Objects	Child likes to direct others’ attention to objects of interest
Cognoa_Cognitive_Behavioral_Social_SharedInterests_SharingToys	Child offers to share toys
Cognoa_Cognitive_Behavioral_Social_Staring_BlankStares	Child tends to stare blankly
Cognoa_Cognitive_Behavioral_Social_Withdrawal_Avoidance	Child has little interest in others
Cognoa_Cognitive_Behavioral_Social_Imitation	Child imitates others’ behavior
Cognoa_Cognitive_ExecutiveFunctioning_Attention_CarelessMistakes	Child tends to make careless mistakes
Cognoa_Cognitive_ExecutiveFunctioning_Attention_FollowingDirections	Child has difficulty following directions
Cognoa_Cognitive_ExecutiveFunctioning_Attention_LosesThings	Child frequently loses belongings
Cognoa_Cognitive_ExecutiveFunctioning_Imagination_WithToys	Child uses creativity in play
Cognoa_Cognitive_ExecutiveFunctioning_Impulsivity_OutOfControl	Child tends to act wild or out of control
Cognoa_Cognitive_ExecutiveFunctioning_Impulsivity_Verbal	Child tends to blurt out the first thing that comes to mind
Cognoa_Cognitive_ExecutiveFunctioning_Memory_CompleteActivities	Child tends to be forgetful in everyday tasks
Cognoa_Cognitive_ExecutiveFunctioning_Memory_ShortTerm	Child has problems with short term memory
Cognoa_Cognitive_ExecutiveFunctioning_Reasoning_LacksFollowThrough	Child tends to lack follow-through
Cognoa_Cognitive_ExecutiveFunctioning_CleaningUpAfterSelf	Child has a tendency to leave behind messes
Cognoa_Cognitive_ExecutiveFunctioning_SloppyWork	Child has sloppy written work
Cognoa_Cognitive_LanguageCommunication_Expressive_SocialChatting	Child is comfortable with social chatting
Cognoa_Cognitive_LanguageCommunication_Speech_UnusualOrOdd	Child has unusual tone or rhythm in speech
Cognoa_Cognitive_LanguageCommunication_Receptive_Conversations	Child can respond to back-and-forth conversations
Cognoa_Motor_Fine_Grip	Child has the ability to grip objects

Twenty-fold cross-validation was performed, and AUCs of 99% (when identifying autism or ADHD) and 99% (for separating autism from ADHD) were observed. This encouraging preliminary result demonstrates the potential utility of the application and the benefit of applying Rosetta instrument mappings to unlock the power of machine-learning in settings that might otherwise not be amenable to such application. Further clinical trial testing should be performed to evaluate how effective such algorithms are when the Rosetta instrument is applied in real world settings.

## Discussion

Our first generation of project Rosetta resulted in a semantic hierarchy that gave us the opportunity to create a minimal set of questions with significant conceptual overlap across multiple childhood behavioral/developmental instruments. This effort to harmonize these instruments relevant to the diagnosis of child developmental disorders including autism and ADHD will be useful for harnessing informatics approaches in order to support child mental, behavioral, and developmental health diagnosis and treatment in the future. Rosetta is not meant to be a new clinical instrument, but rather, a framework that facilitates comparisons and joining operations of disparate data from a variety of medical instruments. This can then be used as an enablement tool for building machine-learning powered assessments that can extract the important features for diagnosing many child behavioral and developmental disorders.

The overlap between existing childhood behavioral/developmental instruments that was created by Rosetta can be used to create a virtual diagnostic tool that covers more patients across various ages and with various conditions in a uniform way that could not be done before. This ability to take in and combine assessment data from any existing instrument through the corresponding mappings allows for the creation of a large, dense dataset that is required for building machine learning algorithms in the development of diagnostic tools as presented in our case study in the “Results” section above.

There are some potential limitations to this project due to the variation between the instruments included in the first generation of Rosetta. There could be a loss of response signals from over-simplification of the question phrasing when creating the Rosetta questions, as well as from mapping existing instrument subject matters to Rosetta questions that are not representative of a particular behavior. Another challenge leading to a loss of response signals comes from the combination of instruments with varying scales of answer choices, as well as the combination of descriptive quality-based answer choices with frequency-based answer choices. Both of these limitations could lead to a misrepresentation of parental responses for particular behaviors.

This work needs to be extended to cover more child behavioral health instruments. Different child behavioral health instruments could potentially expand the mapping to be more representative of other diagnoses that are not well-represented by the eight instruments included in this mapping. This work could also be extended into other diagnostic domains, such as adult behavioral conditions by applying the same concepts to adult checklists and screening tools. Additionally, clinical trial testing should be performed to assess the application of the Rosetta instrument in real world settings across a variety of child behavioral conditions.

## Conclusion

Project Rosetta aims to address challenges regarding the diagnosis of childhood developmental and behavioral conditions through the development of a single, comprehensive semantic hierarchical grouping of concepts that covers a wide array of child behavioral conditions. The resulting Rosetta mapping presented above includes a minimal set of concepts that have diagnostic relevance for many conditions, and can be used as a resource for child mental, behavioral, and developmental health diagnosis and treatment. Rosetta can be used to create more concise instruments across various ages and conditions from the concept-based question bank for both clinical and research use. In addition, the mapping can be used as a framework for joining disparate data in a uniform way to be used in the development of machine learning algorithms and creation of innovative diagnostic tools in the future.

## Data Availability

The training data for the case study mentioned in the methods section is available on demand from the original third party sources: Boston Autism Consortium, Autism Genetic Resource Exchange, Autism Treatment Network, Simons Simplex Collection, and Vanderbilt Medical Center. Declarations

## Abbreviations

AAP: American Academy of Pediatrics
ADHD: Attention-deficit Hyperactivity Disorder
ADI-R: Autism Diagnostic Interview-Revised
ADOS-2: Autism Diagnostic Observation Schedule, Second Edition
ASD: Autism Spectrum Disorder
ASEBA: Achenbach System of Empirically Based Assessment
BASC-3: Behavior Assessment for Children, Third Edition
BRIEF2: Behavior Rating Inventory of Executive Function, Second Edition
BRI: Behavioral Regulation Index
CBCL: Child Behavior Checklist
Conners 3: Conners, Third Edition
CRI: Cognitive Regulation Index
DSM-IV: Diagnostic and Statistical Manual of Mental Disorders, fourth edition
DSM-V: Diagnostic and Statistical Manual of Mental Disorders, fifth edition
ERI: Emotional Regulation Index
ICD: International Classification of Disease
NICHQ: National Initiative for Children’s Healthcare Quality
SRS-2: Social Responsiveness Scale, Second Edition
VADRS: Vanderbilt ADHD Diagnostic Rating Scale

## References

[1] Bitsko RH, Holbrook JR, Robinson LR, Kaminski JW, Ghandour R, Smith C, Peacock G (2016). Health care, family, and community factors associated with mental, behavioral, and developmental disorders in early childhood - united states, 2011-2012. MMWR Morb Mortal Wkly Rep.

[2] Kuhn C, Aebi M, Jakobsen H, Banaschewski T, Poustka L, Grimmer Y, Goodman R, Steinhausen HC (2017). Effective mental health screening in adolescents: Should we collect data from youth, parents or both?. Child Psychiatry Hum Dev.

[3] Rutter M, LeCouteur A, Lord C (2003). Autism Diagnostic Interview - Revised.

[4] Lord C, Rutter M, DeLavore PC, Risi S (2012). Autism Diagnostic Observation Schedule, Second Edition.

[5] Reynolds CR, Kamphaus RW (2015). Behavior Assessment System for Children, Third Edition.

[6] Gioia GA, Isquith PK, Guy SC, Kenworthy L (2015). Behavior Rating Inventory of Executive Function, Second Edition.

[7] Achenbach TM, Rescorla LA (2001). Manual for the ASEBA School-Age Forms & Profiles.

[8] Conners CK (2008). Conners Comprehensive Behavior Rating Scales Manual.

[9] Constantino JN (2012). The Social Responsiveness Scale, Second Edition.

[10] Wolraich ML, Lambert W, Doffing MA, Bickman L, Simmons T, Worley K (2003). Psychometric properties of the vanderbilt adhd diagnostic parent rating scale in a referred population. J Pediatr Psychol.

[11] Dodge M, Kitchin R, Perkins C (2011). The Map Reader: Theories of Mapping Practice and Cartographic Representation.

[12] Goodway JD, Ozmun JC, Gallahue DL (2019). Understanding Motor Development: Infants, Children, Adolescents, Adults.

[13] Henningsen P (2018). Management of somatic symptom disorder. Dialogues Clin Neurosci.

